# Translation of CRISPR Genome Surgery to the Bedside for Retinal Diseases

**DOI:** 10.3389/fcell.2018.00046

**Published:** 2018-05-23

**Authors:** Christine L. Xu, Galaxy Y. Cho, Jesse D. Sengillo, Karen S. Park, Vinit B. Mahajan, Stephen H. Tsang

**Affiliations:** ^1^Jonas Children's Vision Care, Bernard & Shirlee Brown Glaucoma Laboratory, Columbia University, New York, NY, United States; ^2^Department of Ophthalmology, Columbia University, New York, NY, United States; ^3^Frank. H. Netter MD School of Medicine, Quinnipiac University, North Haven, CT, United States; ^4^State University of New York Downstate Medical Center, Brooklyn, NY, United States; ^5^Omics Lab, Department of Ophthalmology, Byers Eye Institute, Stanford University, Palo Alto, CA, United States; ^6^Palo Alto Veterans Administration, Palo Alto, CA, United States; ^7^Department of Pathology & Cell Biology, Columbia University, New York, NY, United States; ^8^Institute of Human Nutrition, College of Physicians and Surgeons, Columbia University, New York, NY, United States

**Keywords:** genome surgery, CRISPR-Cas9, retinal dystrophies, clinical trials, off-target effect

## Abstract

In recent years, there has been accelerated growth of clustered regularly interspaced short palindromic repeats (CRISPR) genome surgery techniques. Genome surgery holds promise for diseases for which a cure currently does not exist. In the field of ophthalmology, CRISPR offers possibilities for treating inherited retinal dystrophies. The retina has little regenerative potential, which makes treatment particularly difficult. For such conditions, CRISPR genome surgery methods have shown great potential for therapeutic applications in animal models of retinal dystrophies. Much anticipation surrounds the potential for CRISPR as a therapeutic, as clinical trials of ophthalmic genome surgery are expected to begin as early as 2018. This mini-review summarizes preclinical CRISPR applications in the retina and current CRISPR clinical trials.

## Background

After the first application of clustered regularly interspaced short palindromic repeats (CRISPR)-mediated gene-editing in human cells in 2013 (Cong et al., 2013; Jinek et al., 2013; Mali et al., 2013), clinical applications of the CRISPR system have become highly anticipated. The CRISPR genome surgery tool is able to edit, delete, insert, activate, repress, epigenetically modify, and induce directed evolution (Doudna and Charpentier, 2014; Hess et al., 2016; Cabral et al., 2017). The growth in the diversity of techniques has potentiated translation from bench to bedside. Conventional gene therapy, which helps restore expression of functional gene products by wild-type gene supplementation, is limited to recessively-inherited diseases and conditions of haploinsufficiency (Sengillo et al., 2016). When a wild-type (WT) gene is supplemented to a cell with a dominantly expressed pathogenic gene, there is no effect on disease progression (Lin et al., 2015). Thus, correction of the pathogenic gene is imperative. As such, the CRISPR system's potential to approach dominantly-inherited conditions is of particular interest (Sengillo et al., 2016; Cabral et al., 2017; Tsai et al., 2018).

While the CRISPR system has great potential for expanding the range of possible treatments for inherited diseases, it cannot be considered a perfect system. Possibly of greatest concern is off-targeting, or unexpected mutations that arise in the process of CRISPR activity (Jamal et al., 2016; Cho et al., 2017; Schaefer et al., 2017). Control of or better understanding of off-targeting should be addressed before CRISPR can be implemented in a broader range of clinical applications.

## Current clinical trials

In October 2016, the first CRISPR clinical trial officially began when Sichuan University recruited the first subject (NCT02793856). So far, 10 CRISPR clinical trials that utilize the CRISPR genome editing tool are registered on ClinicalTrials.gov (Table 1). All of these trials focus on treating various malignancies such as neoplasms and HIV-infection (Table 1). Of the trials that seek to target cancer, six utilize CRISPR-Cas9 to engineer programmed cell death protein (*PD-1*) knockout T cells. PD-1 is a T cell immune checkpoint receptor that interacts with the programmed cell death ligand (PD-L1) on dendritic cells (Su et al., 2016). Normally, when PD-L1 binds to PD-1, the immune response is dampened via peripheral tolerance, which suppresses T-cell proliferation and reduces cytokine production (Fife and Pauken, 2011; Kuol et al., 2018). Cancer cells, however, also express PD-L1. This hijacks the dampening effects of PD-1/PD-L1 interactions. The downside to the currently employed method to block PD-1 receptors—monoclonal antibody delivery—is that it may have unwanted effects on peripheral tolerance by disrupting endogenous dendritic cell/T cell interactions and immune homeostasis (Su et al., 2016). Thus, an *ex-vivo* approach may be favored to inhibit the *PD-1* gene in T cells, which may help avoid problems such as adverse autoimmune effects (Lloyd et al., 2013), yielding a more targeted and efficacious T-cell based therapy.

**Table 1:** CRISPR-Cas9 Clinical Trials as of February 2018.

**NCT ID**	**Diseases targeted**	**Treatment**	**Intervention model**
*NCT03057912*	Human Papillomavirus-Related Malignant Neoplasm	TALEN- or CRISPR-Cas9- mediated disruption of HPV E6/E7 DNA	Parallel Assignment
*NCT02793856*	Metastatic Non-small Cell Lung Cancer	Autologous CRISPR-Cas9-engineered PD-1 knockout-T cells	Parallel Assignment
*NCT02867332*	Metastatic Renal Cell Carcinoma	Autologous CRISPR-Cas9-engineered PD-1 knockout-T cells	Parallel Assignment
*NCT02867345*	Hormone Refractory Prostate Cancer	Autologous CRISPR-Cas9-engineered PD-1 knockout-T cells	Parallel Assignment
*NCT02863913*	Muscle-Invasive Bladder Cancer Stage IV	Autologous CRISPR-Cas9-engineered PD-1 knockout-T cells	Parallel Assignment
*NCT03044743*	Stage IV Gastric Carcinoma; Stage IV Nasopharyngeal Carcinoma; T-Cell Lymphoma Stage IV; Stage IV Adult Hodgkin Lymphoma; Stage IV Diffuse Large B-Cell Lymphoma	Autologous CRISPR-Cas9-engineered PD-1 knockout-T cells	Single Group Assignment
*NCT03166878*	B Cell Leukemia; B Cell Lymphoma	Autologous CRISPR-Cas9-engineered chimeric antigen receptor (CAR)-T cells	Single Group Assignment
*NCT03081715*	Esophageal Cancer	Autologous CRISPR-Cas9-engineered PD-1 knockout-T cells	Single Group Assignment
*NCT03332030*	Neurofibromatosis Type 1; Tumors of the Central Nervous System	n/a: diagnostic test, develop stem cell lines	n/a: Observational
*NCT03164135*	HIV-1-infection	CRISPR-Cas9-engineered CD34+ cells	Single Group Assignment

*n/a, not applicable. Source, ClinicalTrials.gov, accessed February 2018*.

## Preclinical ophthalmic gene-editing

As progress in clinical application of CRISPR genome surgery is made in the field of oncology, it is expected that ophthalmology will soon see clinical advances as well. One of the reasons for this expectation is that ophthalmic genome surgery offers many advantages such as relative immune privileged status due to the blood-retina barrier (Yang et al., 2015). Furthermore, the effects of treatment can be precisely monitored at the resolution of a single cell with non-invasive adaptive optics imaging (Park et al., 2013; Bae et al., 2014; Song et al., 2015, 2018; Yang et al., 2015; Zhang et al., 2015). Another reason for expectation of clinical advances is the demonstrated success in pre-clinical *in vivo* models of retinal dystrophies (Table 2). Table 2 outlines CRISPR-Cas9-mediated successful gene rescue and disease model rescue of various ophthalmic pathogenic genes. While there exists many other successful experiments showcasing the correction of pathogenic genes using CRISPR-Cas9, the pre-clinical *in vivo* models are outlined in this review because *in vivo* models point most directly toward potential clinical applications. Briefly, other applications of CRISPR-Cas9 technology in the retina include disease model generation (Zhong et al., 2015; Arno et al., 2016; Collery et al., 2016) and expanded understanding of disease mechanism (Bassuk et al., 2016; Latella et al., 2016; Yiu et al., 2016; Dong et al., 2017; Kim et al., 2017; Ruan et al., 2017; Yu et al., 2017).

**Table 2:** *In vivo* CRISPR-Cas9 corrections of preclinical retinal disease models.

**Targeted gene & associated disease**	**Disease model**	**CRISPR technique**	**Results**	**References**
*Mertk; arRP*	RCS rat	CRISPR-Cas9 mediated HITI	Gene rescue	Suzuki et al., 2016
*Pde6b; arRP*	*rd1* mouse	CRISPR-Cas9 HDR	Gene rescue	Wu et al., 2016
*Rho; adRP*	Transgenic S334ter rat	CRISPR-Cas9 ablation	Disease model rescue	Bakondi et al., 2016
*RHO; adRP*	P23H *RHO* transgenic mouse	CRISPR-Cas9-induced knock-down	Reduced *RHO* expression	Latella et al., 2016
*Vegfa; CNV, AMD*	laser-induced CNV mouse	Cas9 RNP-mediated gene inactivation	Reduced CNV area	Kim et al., 2017

*arRP, autosomal recessive retinitis pigmentosa; adRP, autosomal dominant retinitis pigmentosa; CNV, choroidal neovascularization; AMD, age-related macular degeneration; HITI, homology-independent targeted integration; HDR, homology-directed recombination; RNPs, ribonucleoproteins*.

One of the new frontiers for CRISPR research in the retina is the modeling and treatment of Leber congenital amaurosis (LCA). LCA, a retinal dystrophy, is the largest cause of childhood blindness worldwide and it can involve up to 20 different genes (Chacon-Camacho and Zenteno, 2015; Maeder et al., 2015). Thus, LCA has been categorized into many subtypes. The gene *CEP290* is implicated in LCA10, which causes 30% of all LCA (Maeder et al., 2015). *CEP290* is very large (containing 54 exons and 7,440 bp in the open reading frame) and thus canonical gene augmentation techniques are difficult due to the limited carrying capacity of the adeno-associated virus (AAV) (Ruan et al., 2017). In May 2015, Maeder et al. reported that CRISPR-Cas9 could fix the cryptic splice site causing the most common mutation in *CEP290* (IVS26 C.2991+1655 A>G mutation) in fibroblasts from LCA10 patients (Maeder et al., 2015). CRISPR-Cas9 fixed the single point mutation—what scientists now call the IVS26 mutation—using two sgRNAs that target sites flanking the aberrant splice site. This allowed *S. aureus* Cas9 to make two double stranded cuts and “repair” the mutant splice site by non-homologous end joining (NHEJ). Expression of the correct *CEP290* transcript due to the removal of the premature stop codon was checked by qRT-PCR.

In February 2017, Ruan et al. successfully removed the IVS26 mutation in *CEP290* from HEK293FT cells carrying the IVS26 mutation (Ruan et al., 2017). They achieved genomic deletion with a pair of sgRNAs, which respectively flanked the splice site in both upstream and downstream directions. After a proof-of-concept *in vitro* experiment, WT mice were used to study *in vivo* applications of CRISPR mediated *Cep290* editing. Wild-type mice were chosen because an animal disease model for LCA10 does not currently exist. Two sgRNAs and *SpCas9* were packaged into separate AAV plasmids in a dual AAV approach, which mediated the deletion of an intronic fragment of the *Cep290* gene in WT mice. The purpose of the dual AAV approach was to circumvent the limitations of AAV carrying capacity.

Translation of *CEP290* CRISPR-Cas9 treatment from bench to bedside may be imminent, because Editas Medicine (Cambridge, MA, USA) recently announced in a press release that clinical trials for CEP290-associated LCA treated with CRISPR-Cas9 technology would begin in 2018 (Taylor, 2017). Further developments in clinical application for other causative mutation retinal dystrophies are highly anticipated as patients suffering from blinding inherited eye disease may gain options for previously untreatable conditions (Doudna and Charpentier, 2014; Sengillo et al., 2016, 2017; Cabral et al., 2017). While expectations are rising, an important aspect of the CRISPR system to consider is undesired off-target mutagenesis, a by-product of CRISPR-Cas9 editing because CRISPR-Cas9 does not have 100% specificity (Tsai and Joung, 2016; Schaefer et al., 2017). However, it should also be acknowledged that there is progress toward understanding off-targeting and reducing off-targeting effects (Tsai and Joung, 2016). One example is Shin et al.'s recent study which demonstrated that the delivery of anti-CRISPR protein AcrIIIA4 in human cells could reduce CRISPR-Cas9 active time. Of note, the team reported reduced off-target editing while on-target CRISPR-Cas9 genome editing remained intact (Shin et al., 2017). Other efforts to address off-targeting include improvements in off-targeting prediction and detection (Tsai et al., 2015; Tsai and Joung, 2016). While the consequences of off-targeting are potentially serious, there is continued development in improving CRISPR-Cas9 specificity (Tsai and Joung, 2016).

## Conclusion

CRISPR genome surgery techniques hold great potential to treat previously unapproachable conditions. While the CRISPR genome surgery system cannot be considered infallible, accelerated progress in recent years has allowed development toward a more specific CRISPR-Cas9. As further developments in CRISPR technology are made to increase on-target accuracy and decrease off-target cleavage, it is expected that the list of CRISPR clinical trials will continue to grow. Especially in ophthalmology, preclinical *in vivo* successes point toward future CRISPR applications in the clinical setting.

## Author contributions

CX and GC contributed equally to this work. CX, GC, JS, and KP wrote and edited manuscript. VM and ST oversaw the writing process.

### Conflict of interest statement

The authors declare that the research was conducted in the absence of any commercial or financial relationships that could be construed as a potential conflict of interest. The reviewer LG and handling Editor declared their shared affiliation.
